# Global longitudinal strain is associated with better outcomes in transcatheter aortic valve replacement

**DOI:** 10.1186/s12872-020-01556-4

**Published:** 2020-06-03

**Authors:** Fadi Al-Rashid, Matthias Totzeck, Nadine Saur, Rolf Alexander Jánosi, Alexander Lind, Amir A. Mahabadi, Tienush Rassaf, Raluca-Ileana Mincu

**Affiliations:** Department of Cardiology and Vascular Medicine, West German Heart and Vascular Center Essen, University Hospital Essen, Medical Faculty, University Duisburg-Essen, 45122 Essen, Germany

**Keywords:** Transcatheter aortic valve implantation, Global longitudinal strain, Clinical outcomes, Echocardiography

## Abstract

**Background:**

Parameters that mark the timing of left ventricular (LV) reverse remodeling following transcatheter aortic valve replacement (TAVR) are incompletely defined. This study aims to identify the dynamics of LV strain derived from speckle tracking echocardiography in a cohort of patients with severe aortic stenosis (AS) who underwent TAVR and its correlation with postprocedural outcomes.

**Methods:**

We selected 150 consecutive patients (82 ± 4 years old, STS score 6.4 ± 6.2) who underwent transfemoral TAVR between 07/2016 and 12/2017 at our tertiary care center**.** All patients were evaluated at baseline, 1 week after TAVR, and 3 months following TAVR.

**Results:**

The global longitudinal strain (GLS) 1 week following TAVR was comparable to that at baseline (− 15,9 ± 4.3 vs − 16.8 ± 4.1; p = NS) but significantly improved at 3 months following TAVR (− 15.9 ± 4.3% vs. -19.5 ± 3.5%; *p* < 0.001). No significant changes in global circumferential strain (GCS) and global radial strain (GRS) were detectable. The ejection fraction was significantly improved 1 week after the TAVR procedure. The baseline GLS correlated directly with the complication rate (R = 0.36, *p* = 0.005). The linear regression analysis showed that the main predictors of the improvement in the GLS at 3 months in our cohort were baseline GRS and GCS.

**Conclusion:**

GLS improves at 3 months after TAVR, while LV ejection fraction does not show a substantial change, signaling an early recovery of LV longitudinal function after the intervention. Additionally, GLS has a direct correlation with the postprocedural outcomes. GLS improvement might emerge as a valuable parameter for a tailored follow-up in TAVR patients.

## Background

Severe aortic stenosis (AS) occurs in 12.4% of people over 75 years of age and represents a substantial burden on health services [1]. Transcatheter aortic valve replacement (TAVR) has emerged as the therapy of choice for patients considered to be at intermediate to high surgical risk or deemed inoperable [2]. Reverse remodeling after TAVR is a complex process determined by left ventricular (LV) pressure, volume and mass reduction. Reverse remodeling has a positive influence on the pathophysiological chain that starts with myocyte hypertrophy and interstitial reactive fibrosis and leads to myocyte atrophy and death, dilatation of the heart, progression through heart failure and increased morbidity and mortality [3–5]. As a consequence, strategies to improve the management of these patients represent a priority.

The echocardiography parameters for defining the reverse remodeling after TAVR are not standardized, and those that could have a prognostic value are not yet established [6–10]. Left ventricular ejection fraction (LVEF), a parameter used in the routine clinical practice to assess LV systolic function, provides limited information about the complex tridimensional ventricular movement during the cardiac cycle. Additional echocardiography techniques, such as speckle tracking echocardiography, allow the analysis of the LV contraction in three different directions—longitudinal, radial and circumferential—and could detect LV remodeling early. LVEF remains preserved until late in the course of the disease for patients with AS, but the early reduction in global longitudinal strain (GLS) signals LV dysfunction and could have prognostic value for these patients. Furthermore, studies have demonstrated that the valvular area and gradients through the stenotic valve do not have prognostic value [11–14].

There is strong evidence of the prognostic value of GLS in heart failure with reduced ejection fraction, acute myocardial infarction or hypertension, pathologies where GLS demonstrated superiority over LVEF for predicting major adverse cardiac events [15–18]. Additionally, GLS is a validated parameter for the detection of therapy-induced cardiomyopathy in patients with neoplasia, by signaling early myocardial dysfunction, months before the reduction in the LVEF [19, 20]. However, its role in the setting of TAVR is incompletely defined.

Although recent studies demonstrated a recovery of LVEF and GLS in patients with severely reduced LVEF after TAVR [21, 22], data regarding the dynamics of LV strain are scarce and inconsistent. This study aims to identify the dynamics of LV strain in a cohort of patients with severe AS who underwent TAVR and its correlation with postprocedural outcomes.

## Methods

### Study population

We enrolled patients with severe symptomatic AS who underwent transfemoral TAVR between 07/2016 and 12/2017 at our tertiary care center in a retrospective manner. The analysis included patients who were treated with one of the following currently CE-approved bioprostheses: Edwards Sapien S3 (Edwards Lifesciences, Irvine, CA, USA) and Medtronic CoreValve or Evolut (Medtronic, Minneapolis, MN, USA). Patients treated with a TAVR bioprosthesis for the management of mitral valve pathology and pure noncalcific aortic regurgitation were excluded.

Patients with symptomatic severe aortic valve stenosis were considered for TAVR if they had a STS Score ≥ 4% or surgery was considered to involve excessive risk due to comorbidities and other risk factors not reflected by the STS Score (e.g., frailty, porcelain aorta, or prior chest radiation). The indication for TAVR in an individual patient was decided upon by consensus of the multidisciplinary heart team (consisting of cardiologists, cardiac surgeons, cardiac anesthetists and physicians from other disciplines whenever needed) according to current guidelines [2]. The exclusion criteria were (a) previous or concomitant replacement of another heart valve, (b) insufficient acoustic window that could prevent a complete echocardiography study, and (c) hemodynamic instability. The study protocol was approved by the Local Ethics Committee (17–7654-BO). All definitions of the clinical endpoints used were in concordance with the Valve Academic Research Consortium 2 definitions [9]**.**

### Study assessment timepoints

All patients were assessed at three different times related to TAVR: within 1 month before TAVR (baseline), within 1 week after TAVR (1 week) and 3 months after TAVR.

### TAVR procedure

TAVR was performed by a multidisciplinary heart team in a hybrid operating room using standard techniques (15, 16) under analgosedation (17) with percutaneous femoral artery access and closure (18). Left ventricular end-diastolic pressure (LVEDP) was measured before balloon valvuloplasty and implantation of a TAVR bioprosthesis.

### Echocardiography and doppler measurements

All subjects underwent standard echocardiographic examination using a commercially available Philips iE-33 ultrasound machine (Philips Electronics, Eindhoven, The Netherlands)**.** Data were transferred to a workstation to be analyzed “offline” using Qlab 10 software (Philips Electronics, Eindhoven, The Netherlands). Standard images of transthoracic echocardiography were obtained from parasternal long- and short-axis views and apical views. Specific acquisitions were performed for tissue Doppler and speckle tracking. The left ventricular ejection fraction was calculated according to Simpson’s method [23], and the on-line E/E’ ratio was calculated as a marker of ventricular filling where E was the early left ventricular filling Doppler wave and E’ was the mean of the lateral and medial mitral ring movement in tissue Doppler imaging [24]. Systolic pulmonary artery pressure (sPAP) was estimated from the pressure gradient between the right ventricle and right atrium added to the right atrial pressure estimated from the inferior vena cava. Right ventricular diameters, area and area change, and tricuspid annular plane systolic excursion (TAPSE) were measured according to the current guidelines [25]. The aortic valve stenosis parameters, such as the planimetric aortic valve area (AVAp), continuity equation aortic valve area (AVAc), and mean LV-Ao gradient (meanPG), were quantified according to the current guidelines [2, 26]. Valvular regurgitations were diagnosed according to previous guidelines [27].

### 2D speckle tracking analysis of LV

To calculate the myocardial end-systolic strain, we used Qlab 10 software. The aortic valve closure was the marker of end-systole. To determine the GLS, the software tracked the full wall region of interest automatically in the three apical views at the end of the diastole and allowed us to adjust the tracking when necessary. We used the 18-segment model to determine the global longitudinal strain. Global radial strain (GRS) and global circumferential strain (GCS) were determined from tracking the short-axis basal, medial and apical views. In this case, a 16-segment model was used [28].

### Statistical analysis

Continuous variables were reported as the mean and standard deviation (SD). Categorical variables were reported as percentages. A paired-samples T test was used to compare continuous variables, while a chi-square test was used to compare categorical variables. In cases of more than two group comparisons, one-way analysis of variance for unpaired parametric, one-way Kruskal-Wallis analysis of variance for nonparametric samples were used or one-way ANOVA for continuous variables, using Bonferroni correction for multiple testing. Pearson’s correlation was used for the association between variables. Cox proportional hazards models were used to calculate hazard ratios and to test for interactions, and stepwise Cox models evaluated the relationship between complication rates and clinical and echocardiographic parameters. Differences with p-values ≤0.05 (2-sided) were considered statistically significant. All analyses were performed using PASW [SPSS] (Version 20, IBM SPSS, Chicago, IL, USA). The authors had full access to the data and take responsibility for their integrity. All authors have read and agreed to the manuscript as written.

## Results

### Baseline and procedural characteristics

A total of 150 patients underwent transfemoral TAVR between 07/2016 and 12/2017. The baseline characteristics of the TAVR cohort are described in Table 1. The study population consisted of elderly patients (mean age 82 ± 4 years) with a mean STS-score 6.4 ± 6.2%. More than 91% presented with NYHA functional class III or IV. The medical history of the patients revealed that the most frequent diseases were stable coronary artery disease, pulmonary hypertension and atrial fibrillation (Table 1). More patients in our cohort had an impaired left ventricular function as determined by GLS (decreased GLS > − 19.7: 83%) as compared to LVEF (decreased LVEF Simpson method ≤52%: 40%). Moreover, 78.6% of patients were treated with self-expandable bioprosthesis (Medtronic CoreValve), while 21.3% were treated with balloon-expandable bioprosthesis (Edwards Sapien). Baseline LVEDP was 20.1 ± 8.6 mmHg. The NYHA functional class improved significantly at 3 months after TAVR.

**Table 1:** Baseline characteristics of the study population

	***n*** = 150
**Age [years]**	82 ± 4
**Female sex % (n)**	52.3 (78)
**STS-score (%)**	6.4 ± 6.2
**NYHA class III/ IV % (n)**	91.3 (137)
**COPD % (n)**	17.3 (26)
**Neurologic dysfunction % (n)**	13.3 (20)
**Chronic renal failure % (n)**	34.7 (52)
**Recent MI % (n)**	17.3 (26)
**Pulmonary hypertension % (n)**	48.7 (73)
**Stable CAD % (n)**	74 (111)
**Previous PCI % (n)**	47.3 (71)
**Previous CABG % (n)**	8.7 (13)
**Peripheral artery disease % (n)**	22 (33)
**Pacemaker % (n)**	13.4 (20)
**Atrial fibrillation % (n)**	45.3 (68)
**LVEF in normal range > 52% %(n)**	63.3 (95)
**Decreased LVEF ≤ 52% %(n)**	39.7 (55)
**Normal GLS ≤ − 19.7% (n)**	17.3 (26)
**Decreased GLS > − 19.7% (n)**	82.6 (124)
**Baseline LVEDP (mmHg)**	20.1 ± 8.6
**Arterial hypertension % (n)**	85.3 (128)
**Diabetes mellitus % (n)**	31.3 (47)
**Adiposity % (n)**	28.2 (42)

*NYHA* New York Heart Association functional classification, *COPD* Chronic obstructive pulmonary disease, *MI* Myocardial infarction, *CAD* Coronary artery disease, *PCI* Percutaneous coronary intervention, *CABG* Coronary-aortic bypass graft surgery, *LVEF* Left ventricular ejection fraction

### Complications and outcome

The most prevalent postprocedural complications in the TAVR cohort were left bundle branch block (32.9%), permanent pacemaker implantation (16%), acute renal failure (14.7%), acute infections (9% pneumonia, 2.6% lower urinary tract infection, 3.8% norovirus or influenza infection), major vascular complications (14.7%), bleeding (1.3% life threatening bleeding, 10% major bleeding), and postprocedural stroke (3.3%). Deep vein thrombosis affected 2.7% of the population, while postprocedural myocardial infarction, cardiac tamponade or intraprocedural death affected 0.7% of the patients.

The survival rate at 30 days was 97.3%, while the survival at 90 days was 96.7%. Five patients died before the 3-month assessment. One 86-year-old male patient died 3 days after the procedure because of cardiogenic shock. One 76-year-old female died 13 days after the procedure due to acute respiratory failure in the context of pulmonary bleeding. One 86-year-old female died 30 days after the procedure from ventricular tachycardia in the context of septic shock. One 71-year-old male died 2 days post procedure from stroke. Finally, one 78-year-old male died from septic shock 2 days post procedure. The one-year survival rate was 96%.

### Conventional echocardiography

LVEF significantly improved from baseline to 1 week after TAVR (50.27 ± 11.13% vs. 52.90 ± 10.58%; *p* < 0.01), but the difference was not highly significant at 3 months (50.27 ± 11.13% vs. 53.51 ± 8.8; p = NS). The LV mass index was slightly decreased without statistical significance. The AVAp, AVAc and meanPG improved significantly after the intervention, as expected. There were no changes between the three assessment timepoints of the sPAP, E’ lateral, E/E’, or TAPSE (Table 2).

**Table 2:** The dynamics of the echocardiography parameters before TAVR (baseline), 1 week and 3 months after TAVR

	Baseline	First week after TAVR	3 months after TAVR	baseline vs. first week (Mean difference [95% CI], p value)	baseline vs. 3 months (Mean difference [95% CI], p value)	p value (ANOVA)
**LVEF [%]**	50.27 ± 11.13	52.90 ± 10.58	53.51 ± 8.8	−2.62 [− 4,11; − 1.14], *p* < 0.01	− 1.43 [− 3.04; 0.17], *p* < 0.05	NS
**LVEDV [ml]**	106 ± 46	113 ± 53	98 ± 543	−6.76 [− 16.30; − 1.41], p = NS	2.42 [−8.24; 13.10], p = NS	NS
**LVESV [ml]**	59 ± 36	58 ± 38	51 ± 32	1.70 [− 3.49; 6.89], p = NS	23.12 [− 18.01; 64.26], p = NS	NS
**LV mass Index [g/m**^**2**^**]**	152 ± 56	158 ± 58	133.42 ± 55.8	−5.58 [− 16.78; 5.06], p = NS	−6.76 [− 16.30; − 1.41], p = NS	NS
**AVAc [cm**^**2**^**]**	0.6 ± 0.16	1.8 ± 0.8	1.52 ± 0.3	− 1.36 [− 1.75; − 0.97], *p* < 0.001	−1.19 [− 1.60; − 0.78], *p* < 0.001	< 0.001
**Mean PG [mmHg]**	42.4 ± 14.5	9.9 ± 4.7	9.33 ± 4.7	32.48 [29.99; 34.97], *p* < 0.001	32.03 [29.05;35.02], *p* < 0.001	< 0.001
**sPAP [mmHg]**	45.7 ± 16.7	47.6 ± 17.1	43.5 ± 12.5	−1.89 [− 5.02; 1.23], p = NS	2.06 [− 1.17; 5.30], p = NS	NS
**E’ lateral**	7.4 ± 2.2	7.5 ± 2.4	7.7 ± 2.7	− 0.17 [− 0.74; 0.48], p = NS	− 0.53 [− 1.29; − 0.22], p = NS	NS
**E/E’**	13.6 ± 5.6	14.6 ± 6.6	14.3 ± 7.8	− 1.02 [− 2.59; 0.55], p = NS	− 1.06 [− 3.28; − 1.15], p = NS	NS
**TAPSE**	17.9 ± 5.2	18.4 ± 4.8	19.7 ± 5.4	−0.5 [− 1.48; 0.39], p = NS	−0.53 [− 1.82; 0.76], p = NS	NS
**GLS [%]**	− 15.9 ± 4.33	− 16.84 ± 4.1	−19.5 ± 3.5	0.93 [− 0.73; 2.60], p = NS	3.84 [2.72; 4.96], *p* < 0.001	< 0.001
**GCS [%]**	−28.03 ± 12	−20.25 ± 10	−30.06 ± 7.2	0.22 [− 4.23; 4.67], p = NS	1.91 [− 2.17; 6.01], p = NS	NS
**GRS [%]**	66.3 ± 30.5	60.25 ± 18.2	64.2 ± 14.02	6.37 [−9.68; 22.43], p = NS	2.2 [− 10.35; 14.75], p = NS	NS

*TAVR* Transcatheter aortic valve replacement, *LVEF* Left ventricular ejection fraction, *LVEDV* Left ventricular end-diastolic volume, *LVESV* Left ventricular end-systolic volume, *AVAc* Continuity equation aortic valve area, *Mean PG* Mean pressure gradient, *sPAP* systolic pulmonary artery pressure, *E*’ Lateral mitral ring movement in tissue Doppler imaging**,***E/E*’ E was the early left ventricular filling Doppler wave and E’ was the mean of the lateral and medial mitral ring movement in tissue Doppler imaging, *TAPSE* Tricuspid annular plane systolic excursion, *GLS* Global longitudinal strain, *GCS* Global circumferential strain, *GRS* Global radial strain

The mitral regurgitation improved 1 week after TAVR, when 35% of patients had moderate mitral regurgitation, compared to 48% of the baseline patients (*p* < 0.05). The 3-month follow-up revealed that 28% of patients had moderate mitral regurgitation (*p* < 0.001). A proportion of 14.6% of patients had severe mitral regurgitation at baseline, compared to 14% at 1 week (p = NS) and 7.5% at 3 months (*p* < 0.05). The rate of postprocedural PVL was 8% in our cohort. The observed PVL were not associated with increased mortality in our cohort.

### 2D speckle tracking echocardiography

**Fig. 1:**
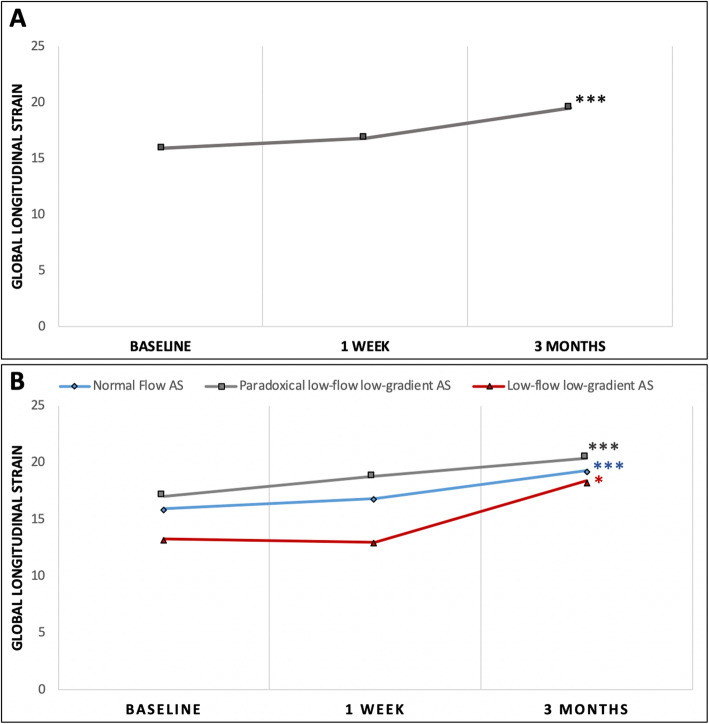
Significant improvement in global longitudinal strain before TAVR and 1 week and 3 months after TAVR for the overall population (A) and three different subgroups (B) . * *p* < 0.05; **p < 0.01; ***p < 0.001. AS = aortic stenosis; TAVR = transcatheter aortic valve replacement

The speckle tracking strain analysis showed that the GLS did not significantly improve 1 week after TAVR (− 15.9 ± 4.33% vs. -16.84 ± 4.1%, p = NS). The 3 months following TAVR assessment showed that GLS significantly improved (Fig. 1) to − 19.5 ± 3.5% (*p* < 0.001). The GCS and GRS were similar between the three assessment points (Table 2).

### Correlations and linear regression analyses

The baseline GLS correlated directly with the complication rate (R = 0.36, *p* = 0.005). The linear regression analysis showed that the main predictors of the improvement of the GLS in our cohort were GRS and GCS at baseline (R^2^ = 0.91, *p* < 0.001). ANOVA showed, that GLS reflects the improvement in ejection fraction significantly better than the 2D LVEF measurement during follow-up time (conventional LVEF p = NS vs. GLS *p* < 0.05). LVEDP correlated significantly with baseline BNP and NTproBNP (R = 0,17, *p* < 0.05 and R = 0.30, *p* < 0.001) and with sPAP (R 0.20, *p* < 0.05).

## Discussion

We performed an analysis of 150 patients who underwent TAVR in our clinic, with a focus on the dynamics of LV strain and its correlation with the clinical outcomes. The main findings of our study are as follows: (i) GLS showed improvement at 3 months following TAVR, although it did not change immediately after TAVR, marking the beginning of the LV reverse remodeling; (ii) baseline GLS correlates with the adverse events following TAVR; and (iii) the main predictors of GLS improvement at 3 months were GRS and GCS at baseline.

Patients with AS have reduced systolic longitudinal LV function despite a normal LVEF because of the gradual development of myocardial fibrosis, predominantly located in the subendocardium [12]. This is why parameters other than LVEF should be used to assess LV systolic function in this category of patients [13, 14]. LV changes after TAVR represent a better model for studying the LV reverse remodeling process compared to surgical aortic valve replacement because factors that could influence the myocardial function during surgery or postoperatively are not present [29]. Many studies focus on the long-term reduction of left ventricular mass after stenotic valve replacement, but the immediate changes reflected in LV strain and their clinical implications are not precisely described [30–33].

Our cohort represents a typical TAVR cohort with older patients with increased operative risk. LVEF significantly improved from baseline to 1 week after TAVR, but the difference was not significant at 3 months. The actual study focuses on the acute changes in LVEF and GLS after TAVR and not on long-term survival data, but we have already shown that LVEF has a prognostic value when it is severely reduced, with patients with LVEF≤40% showing increased mortality after TAVR compared to those with normal and mildly reduced LVEF [34].

The speckle tracking strain analysis showed that the GLS has an ascending trend 1 week after TAVR and improved significantly 3 months after TAVR, irrespective of the AS subtype. The longitudinal function of the LV depends on the subendocardial myocytes, which have a predominantly longitudinal direction, whereas the circumferential and radial function depend on the midwall helicoidal fibers [35]. The longitudinal subendothelial fibers are very susceptible to the reduction in coronary blood flow that occurs in aortic stenosis as a consequence of left ventricular hypertrophy and left ventricular pressure overload [36]. This susceptibility could explain the early recovery of the LV longitudinal function after the replacement of the stenotic aortic valve, in concordance with our findings and data from the literature [37–40]. The increase in GLS after 3 months of follow-up cannot be explained by an increase in LVEF since the LVEF was constant during the follow-up and is most likely explained by the recovery of the longitudinal subendocardial fibers after removing the LV pressure overload through TAVR [41].

We did not demonstrate an improvement in circumferential and radial function; however, our analysis focused on the early changes in echocardiography parameters, and changes in these parameters should be observed at later stages. Nonetheless, there are studies that demonstrated the improvement of all three components of the strain in short-term follow-up on a small sample size or using intraprocedural transesophageal echocardiography, but only changes in GLS persisted during follow-up [42, 43]. The analysis of our cohort data demonstrated that GLS correlates with the complication rate, concordant with other studies where GLS distinguished itself as a prognostic parameter for major adverse cardiac events and was superior to LVEF [18, 22, 44]. Mitral regurgitation, paravalvular leaks or permanent pacemaker implantation are also factors that influence on left ventricular recovery, but we could not observe any significant correlation in our cohort. These results suggest that GLS could be a useful parameter for predicting adverse events after TAVR. To further determine its predictive value for risk stratification, larger prospective studies with long-term outcomes are necessary.

### Limitations

Our study has some limitations that need to be addressed. First, the study is retrospective and descriptive and includes a limited number of subjects, but it reflects real-world clinic activity and can therefore generate new hypotheses. Second, many patients had atrial fibrillation, which could alter the value of strain. Third, technical limitations (e.g. atrial fibrillation, poor image quality) did not permit LV strain analysis in approximately 28% of the patients, and the result could be affected by selection bias. However, there were no significant differences in baseline characteristics or between these two groups. Fourth, the follow-up of the patients was short with of the focus on the acute changes in LV strain, therefore no statement can be made regarding LV recovery over time with a likely LV mass reduction. Long-term survival data showed no influence of baseline strain analysis on long-term outcomes. This is certainly related to the low event rate.

## Conclusion

GLS shows early recovery after TAVR, with significantly higher values at 3 months after the intervention, indicating an early beginning of the reverse remodeling of the LV in patients with aortic stenosis. GLS correlates with adverse events after TAVR and might emerge as a valuable parameter for a tailored follow-up in TAVR patients.

## Supplementary information

**Additional file 1.** Supplementary material - Subgroup analysis.

## Data Availability

The dataset supporting the conclusions of this article is included within the article. The raw data used and/or analysed during the current study are available from the corresponding author on reasonable request.

## Abbreviations

AVAc: Continuity equation aortic valve area
AVAp: Planimetric aortic valve area
AS: Aortic stenosis
GCS: Global circumferential strain
GLS: Global longitudinal strain
GRS: Global radial strain
meanPG: Left ventricular – aortic gradient
LV: Left ventricular
LVEF: Left ventricular ejection fraction
LVEDP: Left ventricular end-diastolic pressure
sPAP: Systolic pulmonary artery pressure
SD: Standard deviation
SVi: Stroke volume index
TAPSE: Tricuspid annular plane systolic excursion
TAVR: Transcatheter aortic valve replacement
