# Supplementary material for: Global longitudinal strain is associated with better outcomes in transcatheter aortic valve replacement
Source: BMC Cardiovasc Disord. 2020 Jun 3;20:267. doi: 10.1186/s12872-020-01556-4 (PMC7268397; doi:10.1186/s12872-020-01556-4)
Supplement: Supplementary file 1 — Additional file 1. Supplementary material - Subgroup analysis. [file 12872_2020_1556_MOESM1_ESM.pdf]

Global longitudinal strain is associated with better outcomes in transcatheter aortic valve replacement

Supplementary material

Fadi Al-Rashid[1], MD, Matthias Totzeck[1], MD, Nadine Saur[1], MS, Rolf Alexander Jánosi[1], MD, Alexander Lind[1], MD, Amir A. Mahabadi[1], MD, Tienush Rassaf[1], MD, FACC, FESC, and Raluca-Ileana Mincu[1], PhD

[1] Department of Cardiology and Vascular Medicine, West German Heart and Vascular Center Essen, University Hospital Essen, Medical Faculty, Germany

[#] Corresponding author
Dr. Fadi Al-Rashid
Department of Cardiology and Vascular Medicine
West German Heart and Vascular Center Essen
University Hospital Essen, Medical Faculty,
University Duisburg-Essen
45122 Essen, Germany
Phone: 0049 201 723 4801
Fax: 0049 201 723 5401
Email: fadi.al-rashid@uk-essen.de

Methods

Subgroup analysis

For the subgroup analysis, we divided the population into three different groups according to the type of AS and defined in accordance with the current guidelines [1, 2]:

- Group 1 included patients with severe AS defined as a valve area <1 cm^2 and a mean gradient >40 mmHg.
- Group 2 included patients with low-flow, low-gradient AS with preserved ejection fraction, also known as the paradoxical low-flow low-gradient AS, which was defined as valve area <1 cm^2, mean gradient <40 mmHg, ejection fraction ≥ 50%, and stroke volume index (SVi) ≤ 35 mL/m2.
- Group 3 included patients with low-flow, low-gradient AS with reduced ejection fraction, defined as valve area <1 cm^2, mean gradient <40 mmHg, ejection fraction < 50%, and SVi ≤ 35 mL/m2.

Results

2D speckle tracking echocardiography

We performed a subgroup analysis of the LVEF and GLS dynamics in three different AS entities (Supplement Table 1). Group 1 included 86 patients with classic AS, group 2 included 31 patients with paradoxical low-flow low-gradient AS and group 3 included 33 patients with low-flow low-gradient AS. While the GLS improved significantly at the 3-month follow-up in all groups, it showed no significant improvement after one week. The LVEF significantly improved one week after the TAVR procedure in patients with classic AS and those with low-flow low-gradient AS (Supplement Table 1).

References

1. Hachicha Z, Dumesnil JG, Bogaty P, Pibarot P: **Paradoxical low-flow, low-gradient severe aortic stenosis despite preserved ejection fraction is associated with higher afterload and reduced survival**. *Circulation* 2007, **115**(22):2856-2864.
2. Baumgartner H, Falk V, Bax JJ, De Bonis M, Hamm C, Holm PJ, Iung B, Lancellotti P, Lansac E, Rodriguez Munoz D *et al*: **2017 ESC/EACTS Guidelines for the management of valvular heart disease**. *Eur Heart J* 2017, **38**(36):2739-2791.

Supplement Table 1. Difference in systolic function between the three study subgroups.

	Baseline	First week after TAVR	3 months after TAVR	baseline vs first week*	baseline vs 3 months*
**GLS**					
**Group 1 (n=86)**	-15.97 ± 2.7	-16.8 ± 3.75	-19.3 ± 4.2	0.82 [-2.04; 3.70], p=NS	3.86 [2.56; 5.17], p<0.001
**Group 2 (n=31)**	-17.05 ± 2.82	-18.83 ± 2.95	- 20.42 ± 2.38	1.77 [-0.24; 3.79], p=NS	3.9 [2.99; 4.81], p<0.001
**Group 3 (n=33)**	-13.35 ± 11.8	-13 ± 7	-18.46 ± 3.03	0.93 [- 2.05 ; 3.79], p=NS	8.02 [1.66; 14.37], p<0.05
**LVEF**					
**Group 1 (n=86)**	52.79 ± 9.73	55.57 ± 9.35	55.23 ± 6.39	-2.77 [-4.91; -0.63], p<0.05	-0.69 [-2.04; 1.01], p=NS
**Group 2 (n=31)**	57.62 ± 4.83	56.33 ± 7.82	56.4± 6.6	1.28 [1.27; 3.84], p=NS	0.65 [-2.84; 4.14], p =NS
**Group 3 (n=33)**	37.46 ± 7.73	43.26 ± 10.07	43.42 ± 11.78	-5.79 [-8.57; -3.02],p<0.001	-6.66 [-12.05; -1.27], p<0.001

*= mean difference, 95% CI, p- value

LVEF = left ventricular ejection fraction; GLS = global longitudinal strain; TAVR = transcatheter aortic valve replacement
